# EBV-positive diffuse large B-cell lymphoma in a patient with primary Sjögren’s syndrome and membranous glomerulonephritis

**DOI:** 10.1186/1471-2369-13-149

**Published:** 2012-11-15

**Authors:** Chang Seong Kim, Yoo Duk Choi, Joon Seok Choi, Eun Hui Bae, Seong Kwon Ma, Soo Wan Kim

**Affiliations:** 1 Department of Internal Medicine, Chonnam National University Medical School, 42 Jebongro, Gwangju, 501-757, South Korea; 2 Department of pathology, Chonnam National University Medical School, Gwangju, South Korea

**Keywords:** Primary Sjögren’s syndrome, Membranous glomerulonephritis, EBV-positive diffuse large B-cell lymphoma

## Abstract

**Background:**

Sjögren’s syndrome is a systemic autoimmune disease in which lymphatic cells destroy the salivary and lacrimal glands. Glomerulonephritis is thought to be a rare occurrence in primary Sjögren’s syndrome. Furthermore, concurrent glomerular involvement and lymphoma in patients with Sjögren’s syndrome has seldom been reported.

**Case presentation:**

A 52-year-old woman with primary Sjögren’s syndrome developed membranous glomerulonephritis and Epstein-Barr virus-positive diffuse large B-cell lymphoma (DLBCL). She was diagnosed with Sjögren’s syndrome based on the dry eyes, dry mouth, positive anti-nuclear antibody test, anti-Ro (SS-A) antibody, salivary gland biopsy, and salivary scintigraphy. Moreover, renal biopsy confirmed the diagnosis of membranous glomerulonephritis. Three months later, her small bowel was perforated with pneumoperitoneum, and the biopsy revealed Epstein-Barr virus-positive DLBCL.

**Conclusions:**

We observed the first case of primary Sjögren’s syndrome associated with Epstein-Barr Virus-positive DLBCL and membranous glomerulonephritis. Because of the possibility of malignancy-associated membranous glomerulonephritis in patients with primary Sjögren’s syndrome, we should be careful and examine such patients for hidden malignancy.

## Background

Sjögren’s syndrome is an autoimmune disease characterized by lymphocytic infiltration of salivary and lacrimal glands, typically presenting with keratoconjunctivitis and xerostomia [1]. Extraglandular manifestations of Sjögren’s syndrome occur in up to a third of patients [2]. Patients with Sjögren’s syndrome can have accompanying interstitial lung disease [3], cutaneous vasculitis [4], peripheral neuropathy [5], hematologic complications [6], and renal involvement. Tubulointerstitial nephritis is the most common renal manifestation of primary Sjögren’s syndrome; whereas, glomerulonephritis is rare in comparison [7]. On the other hand, the most feared complication of primary Sjögren’s syndrome is lymphoproliferative malignancy. The risk of malignant non-Hodgkin’s lymphoma (NHL) is increased in primary Sjögren’s syndrome [6]. Moreover, Epstein-Barr virus (EBV)-positive diffuse large B-cell lymphoma (DLBCL) is rarer and is associated with poorer overall survival than EBV-negative DLBCL. In addition, intestinal involvement and perforation is very rare. Here, we report the case of a 52-year-old woman diagnosed with primary Sjögren’s syndrome associated with EBV-positive DLBCL and membranous glomerulonephritis.

## Case presentation

A 52-year-old woman was admitted to hospital for evaluation of intermittent abdominal tenderness, dry eye, and dry mouth. The dry eye and dry mouth had been present for several years; however, she had not sought medical treatment for these symptoms. She had a history of dilated cardiomyopathy and glaucoma of the left eye. She was being treated with loop diuretics, proton pump inhibitors, and anthocyanosides for glaucoma. In the physical examination, her lower extremities were in an edematous state. Her body temperature was 36.5°C and blood pressure was normal. The results of the laboratory studies were as follows: white blood cell count, 8,600/mm^3^ (neutrophil 73.4%); sodium, 141 mEq/L; chloride, 108 mEq/L; blood urea nitrogen, 15.7 mg/dL; and creatinine, 0.6 mg/dL. The levels of total serum protein, serum albumin, and total cholesterol were 5.5 g/dL, 1.5 g/dL, and 257 mg/dL, respectively. Urinalysis was remarkable for proteinuria (6.3 g/day), 30–49 red blood cells per high-power field, but no other casts. Serologic investigation revealed the presence of anti-nuclear antibody (ANA; 1:80, homogeneous plus speckled pattern), rheumatoid factor (390 IU/mL), and was positive for autoantibody to the Ro (SS-A) antigen (>200 U/mL). However, serology was negative for antibodies against double-stranded DNA, La (SS-B), Sm, ribonucleoproteins, antineutrophil cytoplasmic antibodies, lupus anticoagulant, IgG/IgM anti-cardiolipin, and IgG β2-glycoprotein-1. The C3 levels had decreased to 38 mg/dL (normal, 90–180 mg/dL), whereas C4 and total hemolytic complement 50 levels were within normal ranges at 16 mg/dL and 33.1 U/mL, respectively (normal ranges, 10–40 and 23–46, respectively). Tests for hepatitis B surface antigen, hepatitis C antibodies, cryoglobulins, and human immunodeficiency virus antibodies were negative.

Minor salivary gland biopsy was performed, showing diffuse lymphocytic infiltrations with a focus score of 3 [8]. Moreover, we performed salivary scintigraphy that showed non-visualization in both salivary glands, consistent with the class 4 Schall grading system [9]. Therefore, she was diagnosed with Sjögren’s syndrome based on the dry eyes, dry mouth, positive ANA, anti-Ro (SS-A) antibody, salivary gland biopsy, and salivary scintigraphy, which fulfilled the 2002 American-European consensus classification criteria [10].

**Figure 1:**
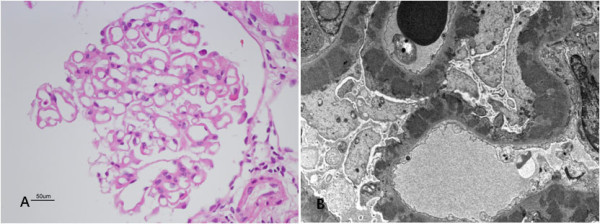
**Percutaneous renal biopsy.** (**A**) On light microscopy, glomeruli show a diffuse thickening of basement membrane with normocellularity (Magnification: x400). (**B**) On electron microscopy, diffuse subepithelial electron dense deposits are easily identified. (Magnification: x2500).

She underwent a percutaneous renal biopsy due to nephrotic syndrome. Glomerular basement membrane thickening and mesangial matrix widening were observed by light microscopy (Figure 1A). In addition, mild tubular atrophy and moderate interstitial fibrosis was noted. Weakly positive staining for IgG, IgA, IgM, and C3 on the outer surface of capillary walls was revealed by immunofluorescent staining. Diffuse subepithelial electron-dense deposit was observed by electron microscopy (Figure 1B). The histopathological findings were consistent with membranous glomerulonephritis [11].

**Figure 2:**
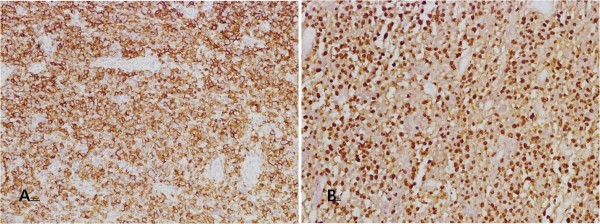
**Small bowel biopsy.** (**A**) The tumor cells have immunoreactivity for CD20. (Magnification: x100). (**B**) The tumor cells have a positive reaction for EBV in situ hybridization. (Magnification: x100).

Abdominal computed tomography (CT) and colonoscopy were performed due to intermittent abdominal tenderness; however, we did not observe any malignancy or bowel perforation at admission except for enteritis and multiple small lymphadenopathies in the mesentery. She had sudden onset of severe abdominal pain 3 months later, and abdominal CT was performed again. Newly developed pneumoperitoneum with peritonitis accompanied by small bowel perforation was observed. An emergency operation and small bowel biopsy were performed. The specimen was 9.5 cm in axial length and had a necrotic and ulcerous (4.5 × 5 cm) lesion. On immunohistochemical examination, we found expression of CD20, CD79a, and BCL-2, but not of CD3, CD10, and BCL-6. The proliferation fraction as determined by Ki-67 was 50%–60%. The majority of tumor cells were positive for EBV by in situ hybridization. Biopsy revealed EBV-positive DLBCL (Ann Harbor classification stage III), as demonstrated in Figure 2. The patient declined treatment with chemotherapy because of poor performance status, low compliance, and poverty. However, she had been receiving intermittent low-dose oral corticosteroid and hydroxychloroquine to treat her primary Sjögren’s syndrome since the diagnosis. There was improvement in proteinuria (1.79 g/day) and sicca symptoms after 1 year of follow-up. One year of follow-up positron emission tomography-CT scan showed a stable disease state for the malignant lymphoma.

## Conclusions

Lymphoma is more common in patients with autoimmune disease, especially those with primary Sjögren’s syndrome [6]. The relative risk of lymphomas in primary Sjögren’s syndrome was found to be 16–44 times higher than in the general population in 2 large case series studies [12,13]. Patients with primary Sjögren’s syndrome have risk factors for progression to lymphoma, such as persistent enlarged parotid glands, splenomegaly, lympadenopathy, palpable purpura, leg ulcers, peripheral nerve involvement, anemia, neutropenia, low-grade fever, low levels of C3 and C4, and mixed cryoglobulinemia [13]. In this case, risk factors for malignant lymphoma included low levels of C3, anemia, and multiple small lymphadenopathies detected by abdominal CT.

EBV-positive DLBCL is characterized by EBV-positive clonal B cell lymphoproliferation that occurs in patients aged over 50 years without any immunodeficiency or prior lymphoma. It is a recently defined subgroup of DLBCL in the 2008 World Health Organization classification of lymphoid neoplasm [14]. The pathogenetic role of the EBV in NHL is circumstantial; however, EBV is able to drive cellular proliferation as a potential carcinogen [15]. In this case, we speculated that this virus assisted in the progression of the malignant lymphoma. Patients with age-related EBV-positive DLBCL are older, have more aggressive clinical features, are rarer (9%) [16], and have poorer overall survival, as compared with EBV-negative DLBCL patients. Lymphoma complicated with Sjögren’s syndrome is usually localized in extranodal areas such as salivary glands, the gastrointestinal tract, the thyroid gland, lung, kidney, or orbit [17]; however, common sites of extranodal involvement in EBV-positive DLBCL are skin, lung, pleural effusion, stomach, and tonsil [18]. Concurrent primary small-bowel involvement and perforation is a very rare clinical manifestation in patients with EBV-positive DLBCL [19], especially in those with accompanying Sjögren’s syndrome.

Interestingly in our case, it is possible that secondary membranous glomerulonephritis was related to primary Sjögren’s syndrome or malignant lymphoma. Clinically significant renal disease is rare in primary Sjögren’s syndrome, but latent involvement is relatively common. Renal abnormalities have been reported at varying rates, from 3% to 67%, in patients with primary Sjögren’s syndrome, according to different studies [20-24]. Nevertheless, Kaufman et al. [2] analyzed 180 case reports of renal involvement in primary Sjögren’s syndrome in a review of the literature; in 89 cases, patients underwent renal biopsies that revealed interstitial nephritis in 49 cases (55%), glomerulonephritis in 33 cases (37%), and both interstitial nephritis and glomerulonephritis in 7 cases (8%). However, renal biopsies been performed in only half of the reported cases; therefore, the true prevalence of membranous glomerulonephritis remains unknown. There have been only 3 previous cases reported of membranous glomerulonephritis and primary Sjögren’s syndrome, to our knowledge [7,25]. From a different point of view, patients with NHL manifest a great variety of glomerular lesions, and several case reports on glomerulonephritis associated with NHL have been published [26-30]. In addition, even if membranoproliferative glomerulonephritis was more common, membranous glomerulonephritis has been reported in 5 of 47 cases (approximately 10%) among glomerulopathies associated with NHL, in a study by Ronco et al. [31]. Furthermore, the onset of proteinuria occurs with the development of lymphoma, or may slightly precede the clinical detection of lymphoma by several months. There have been several case reports where nephritic syndrome was detected prior to the diagnosis of NHL [32-36]. In our case, the patient presented with nephritic syndrome more than 3 months prior to the diagnosis of NHL. In this context, we can tentatively propose the possibility that the secondary membranous glomerulonephritis was related to DLBCL in this case.

Serological abnormalities including those of rheumatoid factor, cryoglobulins, ANA, anti-Ro, and anti-La are common in secondary glomerulonephritis with Sjögren’s syndrome; however, serum complement levels are generally normal unless the patient has associated systemic lupus erythematosus. Goules et al. reported the immunological profile of 10 patients with glomerulonephritis in Sjögren’s syndrome; 2 patients demonstrated low levels of C3, 5 patients had decreased C4 serum levels, and there was 1 patient who had low C3 and normal C4 serum levels. These findings are consistent with those of our case, in which the patient had low C3 and normal C4 levels. The underlying mechanisms are still not completely understood, however, there are varying forms of complement abnormalities in renal involvement accompanying Sjögern’s syndrome. Therefore, further study is needed to better understand complement abnormalities.

Although only a few case reports have described treatment with corticosteroid alone or in combination with cyclophosphamide in primary Sjögren’s syndrome with renal disease, such treatment has generally led to good clinical outcomes [2]. In 1 case report [36], Sjögren’s syndrome complicated with membranous glomerulonephritis resulting from malignant lymphoma resolved after treatment with CHOP (cyclophosphamide, doxorubicin, vincristine, and predinisolone) and radiation. Nevertheless, our patient could not be treated with immunosuppressive therapy or chemotherapy because of her poor general condition. We anticipate that an adequate treatment would be helpful for improvement of the disease.

In conclusion, to the best of our knowledge, we report the first case of primary Sjögren’s syndrome associated with EBV-positive DLBCL and membranous glomerulonephritis. If patients with primary Sjögren’s syndrome show concurrent membranous glomerulonephritis, they should be carefully examined for hidden malignancy, including EBV-positive DLBCL, because of the possibility of malignancy-associated membranous glomerulonephritis.

### Consent

Written informed consent was obtained from the patient for publication of this case report and any accompanying images.

## Abbreviations

ANA: Anti-nuclear antibody; ANCA: Antineutrophil cytoplasmic antibodies; CH50: Complement 50; CT: Computed tomography; DLBCL: Diffuse large B cell lymphoma; EBV: Epstein-Barr virus; NHL: Non-Hodgkin lymphoma; PET-CT: Positron emission tomography-computed tomography.

## Competing interests

The authors declare that they have no competing interests.

## Authors’ contributions

CSK carried out the final preparation of the manuscript. YDC participated in the histological review of biopsies. JSC contributed in the discussion of the conclusions and performed the renal biopsy. EHB and SKM participated in the clinical follow-up of the patient. SWK contributed in the final preparation of the manuscript. All authors read and approved the final manuscript.

## Pre-publication history

The pre-publication history for this paper can be accessed here:

http://www.biomedcentral.com/1471-2369/13/149/prepub
